# Information Theoretical Analysis of Quantum Mixedness in a Finite Model of Interacting Fermions

**DOI:** 10.3390/e27010037

**Published:** 2025-01-06

**Authors:** Diana Monteoliva, Angelo Plastino, Angel Ricardo Plastino

**Affiliations:** 1 UNLP-Comisión de Investigaciones Científicas Provincia de Buenos Aires, La Plata 1900, Argentina; monteoli@fisica.unlp.edu.ar; 2 Instituto de Física La Plata, CCT-CONICET, Universidad Nacional de La Plata, La Plata 1900, Argentina; 3 CeBio-Departamento de Ciencias Básicas, Universidad Nacional del Noroeste Provincia de Buenos Aires (UNNOBA), CONICET, Junin 6000, Argentina; arplastino@unnoba.edu.ar

**Keywords:** Lipkin model, many fermion systems, mixedness-degree, finite temperature, SU2 symmetry

## Abstract

In this study, we utilize information theory tools to investigate notable features of the quantum degree of mixedness ($C_{f}$) in a finite model of *N* interacting fermions. This model serves as a simplified proxy for an atomic nucleus, capturing its essential features in a more manageable form compared to a realistic nuclear model, which would require the diagonalization of matrices with millions of elements, making the extraction of qualitative features a significant challenge. Specifically, we aim to correlate $C_{f}$ with particle number fluctuations and temperature, using the paradigmatic Lipkin model. Our analysis reveals intriguing dependencies of $C_{f}$ on the total fermion number, showcasing distinct behaviors at different temperatures. Notably, we find that the degree of quantum mixedness exhibits a strong dependence on the total fermion number, with varying trends across different temperature regimes. Remarkably, this dependence remains unaffected by the strength of the fermion–fermion interaction (as long as it is non-zero), underscoring the robustness of the observed phenomena. Through comprehensive numerical simulations, we provide illustrative graphs depicting these dependencies, offering valuable insights into the fundamental characteristics of quantum many-body fermion systems. Our findings illuminate the intricate dynamics of the degree of mixedness, a crucial quantum property, with potential implications for diverse fields ranging from condensed matter physics to quantum information science.

## 1. Introduction

One of the key aspects of quantum systems is the concept of quantum mixedness, which quantifies the degree to which a quantum state is a mixedness of different possible states, rather than a pure state. This property is crucial in various fields, from condensed matter physics to quantum information science, as it influences the behavior and characteristics of quantum systems.

The quantum notion of mixedness [1,2,3,4,5], in the context of many-body quantum systems [6,7,8,9], becomes particularly intriguing. Many-body systems, composed of interacting particles, exhibit complex behaviors that arise from the interplay of their individual components [6]. The degree of quantum mixedness ($C_{f}$) in such systems can reveal significant insights into their underlying physics. However, studying these systems poses substantial challenges, especially when dealing with realistic models that require the diagonalization of extremely large matrices [6,7,8,9].

To address these challenges, simplified models that capture the essential features of more complex systems are often employed. One such model is the Lipkin–Meshkov–Glick (LMG) model [6,7,8,9], which has been extensively used to study various aspects of interacting fermions. The LMG model provides a manageable yet insightful framework to explore the qualitative features of quantum many-body systems, making it an ideal choice for investigating the dependencies of quantum mixedness [6,7,8,9].

In this study, we utilize the LMG model to analyze the quantum degree of mixedness in a finite system of *N* interacting fermions. By correlating $C_{f}$ with particle number fluctuations and temperature, we aim to uncover the fundamental dependencies and behaviors of quantum mixedness in many-body fermion systems. Our investigation reveals intriguing trends in $C_{f}$ across different temperatures and total fermion numbers, highlighting the robustness of these phenomena against variations in the fermion–fermion interaction strength.

Through comprehensive numerical simulations, we provide a detailed theoretical framework and illustrative graphs that depict these dependencies. Our findings not only enhance the understanding of quantum mixedness in many-body systems but also have potential implications for various applications, from improving models of atomic nuclei to advancing quantum computing techniques.

The significance of this study lies in its ability to bridge the gap between simplified theoretical models and the complex reality of many-body quantum systems. By elucidating the behavior of quantum mixedness, we contribute to the broader comprehension of quantum mechanics and its applications, paving the way for future research in both theoretical and practical domains.

### 1.1. Our Present Protagonist: The Mixedness Notion

Mixedness plays an essential role in quantum tasks for various reasons. We list some of them.

- Understanding Quantum Correlations and Entanglement: The degree of mixedness provides insights into the quantum correlations and entanglement within a system. By studying the degree of mixedness, researchers can gain a deeper understanding of how entanglement is distributed and how it can be manipulated within a system.
- Characterizing Quantum States: The degree of mixedness helps characterize the purity of quantum states. Pure states, which have zero mixedness, are ideal for many quantum processes, while mixed states, which have non-zero mixedness, can result from decoherence and other environmental interactions. Understanding the mixedness can help in designing strategies to preserve quantum coherence and improve the performance of quantum devices.
- Analyzing Thermodynamic Properties: The degree of mixedness is linked to the thermodynamic properties of quantum systems. It can provide information about phase transitions, thermalization processes, and the overall behavior of a system at different temperatures. This is particularly important in understanding quantum statistical mechanics and the thermodynamics of small systems where quantum effects are significant.
- Exploring Quantum-to-Classical Transition: By studying how the degree of mixedness changes with various parameters, such as temperature and particle number, researchers can gain insights into the quantum-to-classical transition. This transition is crucial for understanding how classical behavior emerges from quantum systems, a fundamental question in quantum mechanics.
- Implications for Quantum Simulations: In quantum simulations of complex systems, knowing the degree of mixedness can help validate the accuracy of the simulations. It ensures that the simulated quantum states accurately reflect the intended physical systems, particularly when simulating open systems that interact with their environment.
- Overall, scrutinizing the features of the degree of mixedness in a quantum system is essential for advancing our understanding of quantum mechanics, improving quantum technologies and exploring new frontiers in quantum research. It provides a critical link between theoretical concepts and practical applications, enabling researchers to harness the full potential of quantum systems.

### 1.2. Our Purposes Here

The degree of quantum mixedness is influenced by several factors, including the total number of fermions and the temperature of the system [10,11,12,13,14]. Understanding how these factors **interact and affect the mixedness-degree** $C_{f}$ is crucial for gaining deeper insights into the quantum dynamics of many-body systems. Through comprehensive numerical simulations, we investigate these dependencies and present our findings through illustrative graphs. Our results reveal that the degree of mixedness is strongly dependent on the total fermion number, with distinct trends emerging across different temperature regimes. Interestingly, this dependence seems to be unaffected by the strength of the fermion–fermion interaction, provided it is non-zero. Hopefully, our results will highlight the fundamental nature of the observed phenomena.

## 2. The SU2-Angular Momentum Lipkin Quasi-Spin Formalism [6,7,8]

We deal with *N* fermions and call Ω=N/2; that is, the Lipkin model consists of N=2Ω fermions that occupy two different N-fold degenerate single-particle (sp) energy levels. The two levels are separated by an energy gap ϵ. This entails 4Ω s.p. micro states. Two quantum numbers (called μ and *p*) are linked to a single micro state. The first one, μ, takes the values μ=−1 (lower level) and μ=+1 (upper level). The remaining quantum number is denoted the quasi spin *p* pertaining to the 2N-fold degeneracy. The pair *p*, μ is viewed as a ”site”, which can be occupied (by a fermion) or be empty. Lipkin fixes

(1) N=2J.

Here, J is a sort of angular momentum. Lipkin [7] uses special angular momentum operators called quasi-spin ones. These are

(2) $$J_{z}=\sum_{p,\mu }\mu \;C_{p,\mu }^{+}C_{p,\mu },$$

(3) $$J_{+}=\sum_{p}C_{p,+}^{+}C_{p,-},$$

(4) $$J_{-}=\sum_{p}C_{p,-}^{+}C_{p,+},$$

together with the Casimir operator

(5) $$J^{2}=J_{z}^{2}+\frac{1}{2}\left(J_{+}J_{-}+J_{-}J_{+}\right).$$

The eigenvalues of $J^{2}$ take form J(J+1) and the Lipkin Hamiltonian reads (*v* is a coupling constant)

(6) $$H=\epsilon J_{z}+\frac{v}{4}\left(J_{+}^{2}+J_{-}^{2}\right).$$

For the Lipkin Hamiltonian, we have the matrix [7]

(7) $$\begin{array}{ccc} \langle n^{\prime }|H_{L}|n\rangle & = & \left\{\frac{N}{2}-n+1-\left(Nn-\frac{N}{2}-n^{2}+2n-1\right)\omega \right\}\delta_{n^{\prime },n}-\\ & & -\frac{v}{2}\sqrt{(N-n)(N-n+1)(n+1)n}\;\delta_{n^{\prime },n+2}\\ & & -\frac{v}{2}\sqrt{\left(N-n^{\prime }\right)\left(N-n^{\prime }+1\right)\left(n^{\prime }+1\right)n^{\prime }}\;\delta_{n^{\prime },n-2} \end{array}$$

with n=0,1,…,N for J=N/2. Numerical diagonalization yields energy eigenvalues $E_{n}\left(v,J\right)$ for our Hamiltonian. These eigenvalues are needed to build the partition function *Z* in the canonical ensemble [13,14].

All thermal quantities of interest are deduced from the partition function *Z* [13,14]. We construct *Z* using probabilities assigned to the models’ microscopic states. Their energies are $E_{i}$ [13,14]. Some important macroscopic quantifiers are computed as in [13,14]. These indicators, together with *Z*, derive from the canonical probability distributions [13,14]. $P_{n}\left(v,J,\beta \right)$. β is the inverse temperature. The pertinent expressions are given in [13,14]. If we call the mean energy *U* and the free energy *F*, we have

(8) $$\begin{array}{ccc} P_{n}\left(v,J,\beta \right) & = & \frac{1}{Z(v,J,\beta )}e^{-\beta E_{n}\left(v,J\right)} \end{array}$$

(9) $$\begin{array}{ccc} Z(v,J,\beta ) & = & \sum_{n=0}^{N}e^{-\beta E_{n}\left(v,J\right)} \end{array}$$

(10) $$\begin{array}{ccc} U(v,J,\beta ) & = & \langle E\rangle =-\frac{\partial lnZ(v,J,\beta )}{\partial \beta }\\ & = & \sum_{n=0}^{N}E_{n}\left(v,J\right)P_{n}\left(v,J,\beta \right)\\ & = & \frac{1}{Z(v,J,\beta )}\sum_{n=0}^{N}E_{n}\left(v,J\right)e^{-\beta E_{n}\left(v,J\right)} \end{array}$$

(11) $$\begin{array}{ccc} S(v,J,\beta ) & = & -\sum_{n=0}^{N}P_{n}\left(v,J,\beta \right)\ln\left[P_{n}\left(v,J,\beta \right)\right] \end{array}$$

(12) $$\begin{array}{ccc} F(v,J,\beta ) & = & U(v,J,\beta )-T\;S(v,J,\beta ). \end{array}$$

The thermal quantifiers above provide much more information than the one obtained via just the quantum resources of zero temperature *T* [13,14]. As stated above, taking a low enough *T*, our quantifiers above yield a good representation of the T=0 scenario [13,14]. Below, we adopt the high enough β=20 value.

### The State’s ρ Degree of Mixedness $C_{f}$

The concepts of purity and degree of mixedness are fundamental in quantum mechanics and play a crucial role in describing the behavior of quantum systems. They are particularly relevant in the study of quantum information, quantum computation, quantum entanglement, and quantum measurements. The distinction between pure states and mixed states allows for a comprehensive understanding of the coherence, superposition, and statistical behavior of quantum systems.

As is well known in quantum mechanics, the degree of mixedness $C_{f}$ of a given state represented by ρ is given by

(13) $$C_{f}=1-Tr\rho^{2}=1-\sum_{n}P_{n}^{2},$$

where, $Tr\rho^{2}$ is the so called ”Purity” $P_{y}$. Note that we have $C_{f}=0$ and $P_{y}=1$ for pure states. $C_{f}$ is a very important quantity for us here.

In probability terms, we have $P_{y}=\sum_{n=0}^{N}{\left(P_{n}\left(v,J,\beta \right)\right)}^{2}$ and $C_{f}=S_{2}=1-P_{y}^{2}$.

## 3. First Results

### 3.1. $C_{f}$ Versus Temperature T

**Figure 1:**
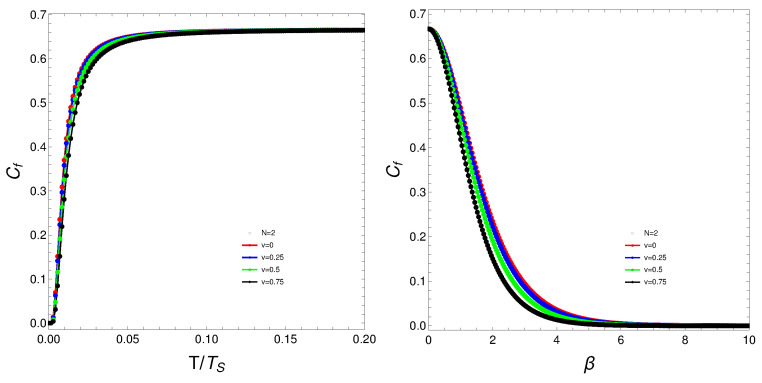
(**Left**) $C_{f}$ versus $T/T_{S}$ where $T_{S}$ is that temperature for which the values of $C_{f}$ stabilize. (**Right**) $C_{f}$ versus β. For both graphs, *N* is fixed at N=2, and each curve corresponds to a different *v*-value.

**Figure 2:**
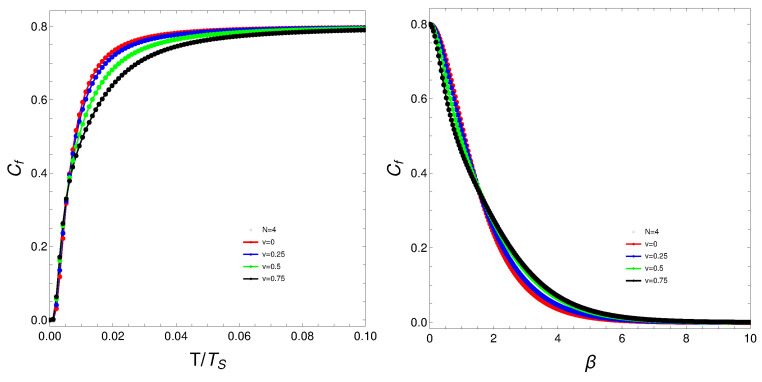
(**Left**) $C_{f}$ versus $T/T_{S}$, where $T_{S}$ is the temperature for which the values of $C_{f}$ stabilize. (**Right**) $C_{f}$ versus β. For both graphs, *N* is fixed at N=4, and each curve corresponds to a different *v*-value.

**Figure 3:**
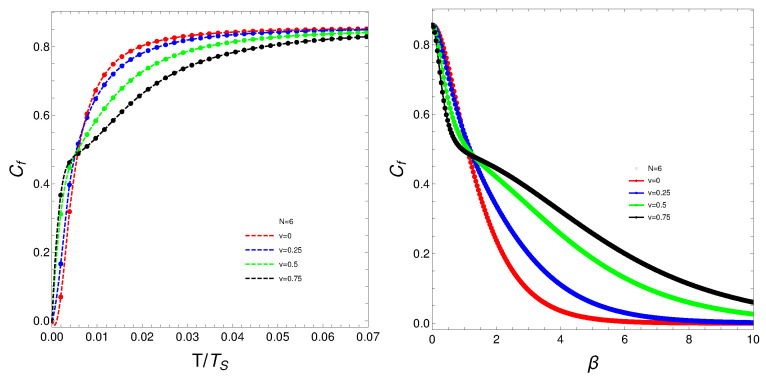
(**Left**) $C_{f}$ versus $T/T_{S}$, where $T_{S}$ is the temperature for which the values of $C_{f}$ stabilize. (**Right**) $C_{f}$ versus β. For both graphs, *N* is fixed at N=6, and each curve corresponds to a different *v*-value.

**Figure 4:**
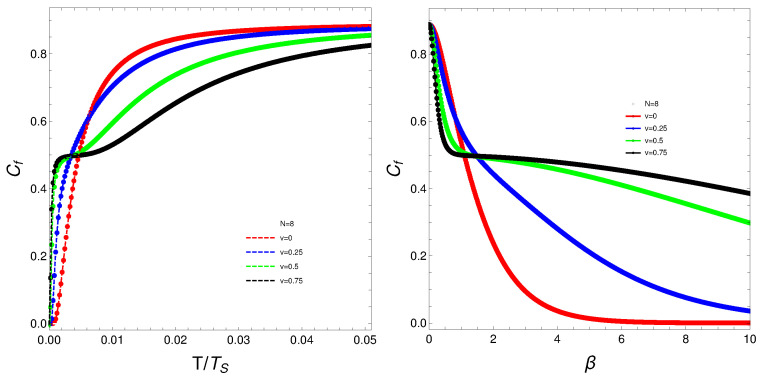
(**Left**) $C_{f}$ versus $T/T_{S}$, where $T_{S}$ is the temperature for which the values of $C_{f}$ stabilize. (**Right**) $C_{f}$ versus β. For both graphs, *N* is fixed at N=8, and each curve corresponds to a different *v*-value.

**Figure 5:**
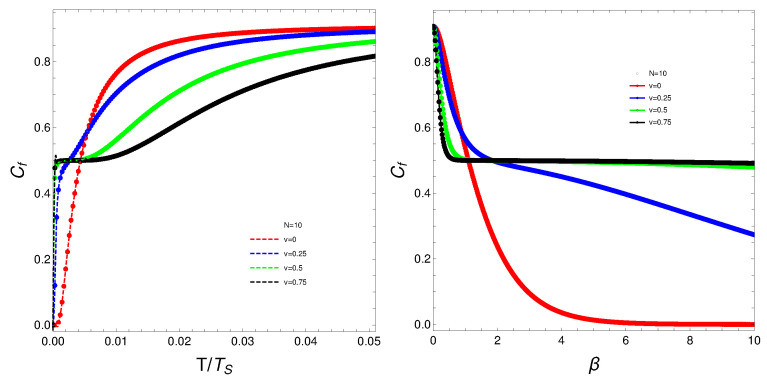
(**Left**) $C_{f}$ versus $T/T_{S}$, where $T_{S}$ is the temperature for which the values of $C_{f}$ stabilize. (**Right**) $C_{f}$ versus β. For both graphs, *N* is fixed at N=10, and each curve corresponds to a different *v*-value.

**Figure 6:**
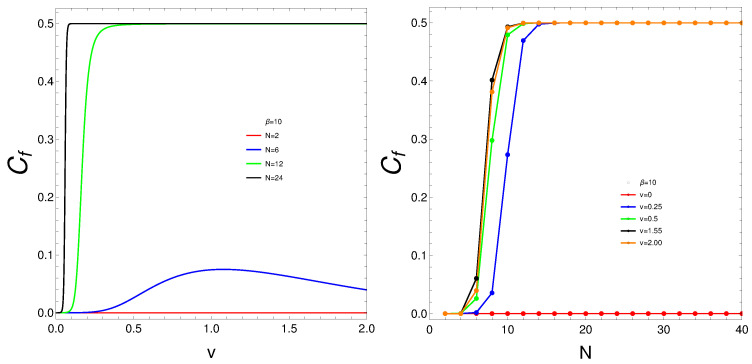
(**Left**) $C_{f}$ versus *v* for different *N*-values. There seems to be a kind of phase transition for *N* large enough. (**Right**) $C_{f}$ versus *N* for different *v*-values. Even for small *N* the mixing abruptly grows. For both graphs β=10.

**Figure 7:**
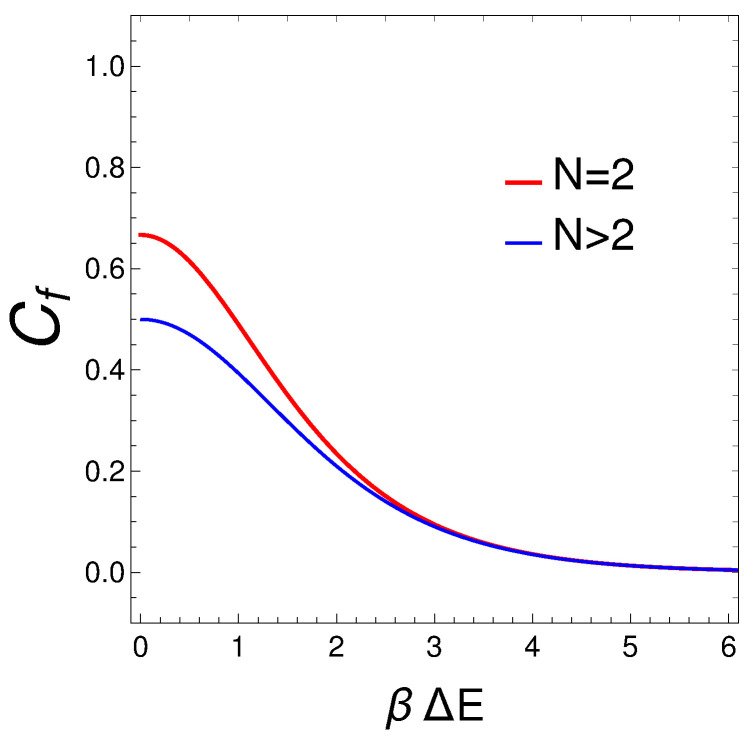
$C_{f}$ versus βΔE for N=2 (red curve) and several other fermion numbers (4, 6, 8, 10) in the other curve, on which they coalesce.

The quantity $C_{f}$ will be the protagonist of Figures 1–7. Strong changes in the system’s dynamics emerge as *N* grows. We plot below the mixing $C_{f}$ versus *T* and versus the inverse temperature β. There seems to be a critical temperature $T_{S}$ at which $C_{f}$ stabilizes itself and ceases growing with *T*. It is clear that when *N* reaches the value six, the system’s behavior strongly changes as multiple artifacts arise, whereas for N<6, the mixing grows smoothly with *T*. In somewhat more detail, we can make the following assertion.

The analysis of the degree of quantum mixedness ($C_{f}$) as a function of temperature (*T*) reveals significant insights into the system’s thermodynamic behavior. As the number of particles (*N*) increases, pronounced changes in the system’s dynamics become evident. In the provided plots of $C_{f}$ versus *T* and versus the inverse temperature (β) [Figure 1, Figure 2, Figure 3, Figure 4 and Figure 5], a critical temperature, $T_{S}$, emerges where $C_{f}$ stabilizes and ceases to grow with increasing *T*. This indicates a phase transition-like behavior, where the system transitions from one state to another, exhibiting stability in the degree of mixedness beyond $T_{S}$. This critical temperature suggests a threshold above which the system reaches a state of equilibrium regarding its mixedness. For N≥6, the system’s behavior changes markedly, introducing multiple artifacts and complexities. This indicates a possible shift in the underlying dynamics or interactions within the system as it scales. In contrast, for N<6, $C_{f}$ exhibits a smoother and more predictable growth with *T*, suggesting a more straightforward relationship between temperature and quantum mixedness in smaller systems. The observed artifacts for larger *N* could be attributed to increased interaction complexities and emergent phenomena that are not present in smaller systems. These artifacts might reflect underlying phase transitions, resonance effects, or other collective behaviors that only manifest at higher particle numbers. Overall, the behavior of $C_{f}$ with temperature highlights the intricate dynamics of many-body quantum systems and underscores the importance of considering particle number effects when analyzing thermodynamic properties. These findings provide valuable insights into the quantum mechanical properties of the system and have potential implications for understanding more complex, realistic models in quantum physics.

**Figure 8:**
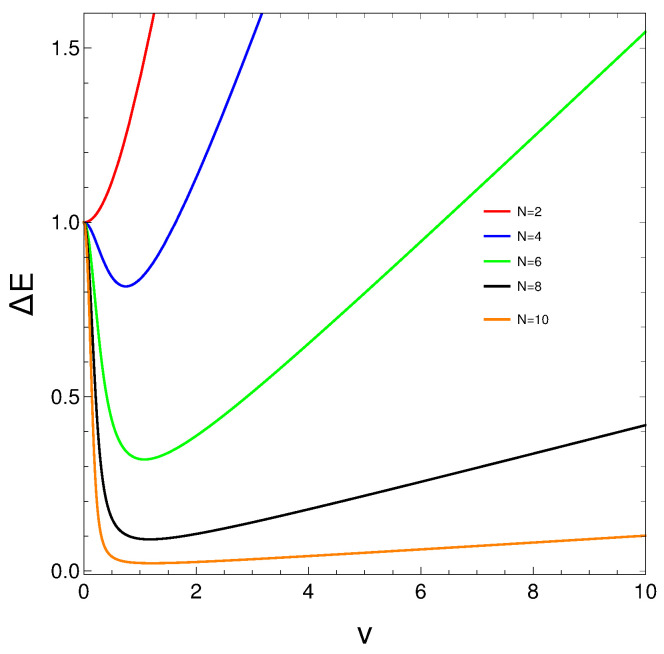
ΔE versus *v* for different *N*s.

**Figure 9:**
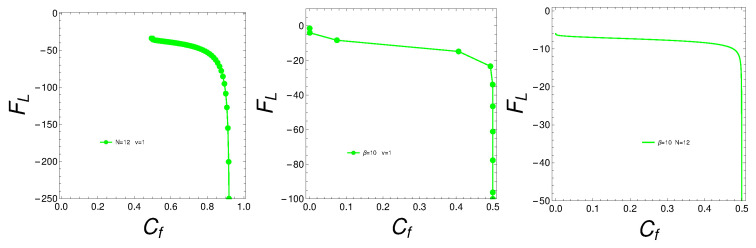
Lipkin free energy $F_{L}$ versus $C_{f}$ in various conditions described in the text.

What do we learn from these graphs? That the mixing strongly depends not only on the temperature but also, and in a very strong fashion, on the fermion number and the coupling constant. In summary, the degree of quantum mixing in the Lipkin model is a multifaceted phenomenon influenced by temperature, fermion number, and the coupling constant. Each of these factors contributes to the overall behavior of the system, determining how the quantum states are occupied and mixed. Understanding these dependencies is crucial for analyzing the dynamics and properties of many-fermion systems within the framework of the Lipkin model. Further analysis on these issues follows below. See Figures 6–9.

### 3.2. $C_{f}$ Changes as Plotted Versus v or Versus N

The behavior of $C_{f}$ in these circumstances is depicted in Figure 6. We again detect a critical *v* at which stability is reached. Same for *N*. Here, however, we see quite abrupt jumps in the mixedness-degree as the coupling constant grows for *N* large enough or for *v* finite.

Let us reflect upon the two above graphs. The interaction between fermions acts as a perturbation that drives the system through a quantum phase transition. Before the interaction is introduced, the system may be in a non-mixed, ordered phase with a degree of mixing at zero.

- As soon as the interaction is turned on, it reaches a critical threshold where the system can no longer maintain this ordered phase, leading to an abrupt jump to a mixed phase with a degree of mixing at 0.5. This critical interaction threshold marks a point where the system’s energy landscape changes significantly.
- At this critical point, the system’s ground state undergoes a reconfiguration. The abrupt jump in the degree of mixing suggests that the system transitions from a non-mixed state to a highly mixed state in which the particles are now in a superposition of states.
- This reconfiguration minimizes the system’s free energy under the new interaction regime, as we see below, leading to a more stable state with increased quantum coherence and entanglement among the fermions.

- The jump to a mixing of 0.5 indicates a sudden onset of quantum coherence. The system achieves a new equilibrium where the quantum states are coherently mixed, resulting in an optimal balance of energy.

- The high degree of mixing implies that the system has transitioned to a state where fermions are delocalized and strongly correlated, maximizing the entropy, as we confirm below, Thus, introducing fermion interactions induces strong correlation effects that are not present in the non-interacting system. These correlations enhance the mixing of quantum states, leading to a robust mixed phase.

- The interactions cause the particles to collectively behave in a way that drastically alters the macroscopic properties of the system, reflected in the sudden change in the degree of mixing. The abrupt change in the degree of mixing highlights the robustness of the quantum phase transition. It demonstrates that even a small interaction can lead to significant changes in the system’s macroscopic properties when the number of particles is large enough.

- This robustness indicates that the system’s behavior is dominated by collective effects rather than individual particle properties, a hallmark of many-body quantum systems. We can make here more elaborate reflections by analyzing and expanding on these points:

1. Interaction-Induced Phase Transition: The interaction between fermions acts as a perturbation that drives the system through a quantum phase transition. Initially, in the absence of interactions, the system is in a non-mixed, ordered phase with zero degree of mixing. When the interaction is introduced, it reaches a critical threshold where the system transitions abruptly to a mixed phase with a degree of mixing around 0.5. This critical interaction threshold signifies a significant change in the system’s energy landscape.
2. Ground State Reconfiguration: At the critical point, the system’s ground state undergoes a reconfiguration. The sudden jump in the degree of mixing from 0 to 0.5 suggests a rapid transition from a non-mixed to a highly mixed state, indicating that particles are now in a superposition of states. This reconfiguration minimizes the system’s free energy under the new interaction regime, leading to a more stable state characterized by increased quantum coherence and entanglement among the fermions.
3. Quantum Coherence and Entanglement: The abrupt onset of a high degree of mixing (0.5) indicates the emergence of significant quantum coherence. The system achieves a new equilibrium where quantum states are coherently mixed, optimizing energy balance. This high degree of mixing implies that fermions are delocalized and strongly correlated, leading to maximized entropy. The introduction of fermion interactions induces strong correlation effects that enhance the mixing of quantum states, resulting in a robust mixed phase.
4. Collective Behavior and Macroscopic Property Changes: The interactions cause particles to behave collectively, dramatically altering the system’s macroscopic properties. The sudden change in the degree of mixing reflects the robustness of the quantum phase transition. It demonstrates that even small interactions can lead to substantial changes in macroscopic properties when the particle number is sufficiently large. This robustness highlights that the system’s behavior is governed by collective effects rather than individual particle properties, a hallmark of many-body quantum systems.
5. Implications for Many-Body Quantum Systems: The findings underscore the importance of collective behaviors in many-body quantum systems. The interaction-driven transition to a mixed phase with high quantum coherence and entanglement illustrates how many-body effects can dominate system dynamics. These results are crucial for understanding quantum phase transitions and the emergence of macroscopic quantum properties in interacting fermion systems.
6. In summary, the analysis presented in the remarks provides a comprehensive explanation of the effects of fermion interactions on the system’s thermodynamic and quantum mechanical properties. The critical threshold, abrupt jumps in mixing, and the resulting coherent states offer valuable insights into the behavior of many-body quantum systems and their phase transitions. These findings have significant implications for theoretical models and practical applications in quantum physics.

## 4. Connection Between the Degree of Mixedness and the Differences Between Energy Levels

We discuss now results plotted in Figures 7–9 below. It is shown in reference [15], Figure 1, that the energy difference ΔE between those for the ground state energy and the first excited state diminishes as *v* grows, facilitating mixing. Diminishing the energy difference between the first excited state and the ground state facilitates mixing in the system due to increased thermal population and enhanced quantum mechanical coupling. In a system at thermal equilibrium, the population of states is governed by the Gibbs canonical distribution $P_{i}\propto \exp E_{i}/k_{BT}$, where $E_{i}$ is the energy of state *i*, $k_{B}$ is the Boltzmann constant, and *T* is the temperature.

When the energy difference ΔE between the ground state and the first excited state decreases, the exponential factor exp($\Delta E/k_{BT}$) increases. This means that at a given temperature, more particles are thermally excited to the first excited state. Furthermore, there is increased occupancy, as a higher thermal population in the first excited state leads to a greater number of particles available to transition between these states, facilitating interactions and mixing. Additionally, the probability of quantum transitions between states depends on the overlap of their wavefunctions and the energy separation. A smaller energy gap can enhance the coupling between the ground state and the first excited state, increasing the transition rate between these states.

Let us see what happens for N=2, involving diagonalization of a 3 × 3 matrix. We look for an analytical expression for ΔE as a function of *v* and the plot $C_{f}$ versus ΔE for several values of *v*. In diagonalizing the Hamiltonian $H_{L}$ for N=2, the energies are

(14) $$\begin{array}{ccc} E_{0} & = & -\sqrt{1+v^{2}} \end{array}$$

(15) $$\begin{array}{ccc} E_{1} & = & 0 \end{array}$$

(16) $$\begin{array}{ccc} E_{2} & = & \;\;\sqrt{1+v^{2}} \end{array}$$

Accordingly $\Delta E\left(v\right)=\sqrt{1+v^{2}}$ and ΔE(v=0)=1. The states’ probabilities are

(17) $$\begin{array}{ccc} P_{0} & = & \frac{1}{Z}e^{\beta \sqrt{1+v^{2}}}=\frac{1}{Z}e^{\beta \Delta E} \end{array}$$

(18) $$\begin{array}{ccc} P_{1} & = & \frac{1}{Z}e^{0}=\frac{1}{Z} \end{array}$$

(19) $$\begin{array}{ccc} P_{2} & = & \frac{1}{Z}e^{-\beta \sqrt{1+v^{2}}}=\frac{1}{Z}e^{-\beta \Delta E}, \end{array}$$

with $Z=1+e^{\beta \sqrt{1+v^{2}}}+e^{-\beta \sqrt{1+v^{2}}}=1+2\cosh\left(\beta \sqrt{1+v^{2}}\right)=1+2\cosh\left(\beta \Delta E\right)$. As a consequence, we have for $C_{f}$

(20) $$\begin{array}{ccc} C_{f} & = & 1-\sum_{n}P_{n}^{2}=1-\frac{1+2\cosh\left(2\beta \Delta E\right)}{{\left[1+2\cosh\left(\beta \Delta E\right)\right]}^{2}}, \end{array}$$

which is plotted in Figure 7 ($C_{f}$ versus βΔE) for several numbers of fermions. N=2 is a special instance. Other varieties of *N* exhibit collapse into a single curve. As expected, the degree of mixing increases as βΔE diminishes.

We can easily think on reasons why, in an interacting many-fermion system, the degree of quantum mixing, or mixedness, tends to increase as the separation between energy levels diminishes. This phenomenon can be understood through several interrelated concepts in quantum mechanics and statistical mechanics.

1. Energy Level Density and Quantum States: As the separation between energy levels decreases, the density of available quantum states increases. When energy levels are closely spaced, fermions have a larger number of states to occupy within a small energy range. This increased state density enhances the probability of transitions between states, leading to greater mixing of quantum states.

2. Thermal Excitations: At low temperatures, fermions typically occupy the lowest available energy states due to the Pauli exclusion principle. However, as the energy level separation decreases, even small thermal excitations can cause fermions to transition between states. This results in a higher degree of occupation of excited states, contributing to quantum mixing.

3. Interaction-Induced Mixing: Interactions between fermions can lead to the hybridization of states, where the eigenstates of the system become superpositions of non-interacting states. When energy levels are closely spaced, interactions more readily cause mixing because the energy required to couple states is lower. This leads to an increased degree of quantum mixedness as interactions redistribute the fermions among the available states.

4. Quantum Fluctuations: In systems with closely spaced energy levels, quantum fluctuations become more significant. These fluctuations can induce transitions between states, further enhancing quantum mixing. The reduced energy gap means that even small perturbations (thermal or quantum) can cause changes in the occupation of states.

Now, let us take a look at the behavior of ΔE versus *v* for different *N*-values in Figure 8.

ΔE grows monotonously only for N=2. We detect a local minimum for N>2. The minima features for ΔE are clearly *N*-dependent.

## 5. Free Energy F Versus $C_{f}$

We pass to analyze the free energy behavior in relation to the mixedness degree. We plot *F* versus $C_{f}$ considering either β, *N*, or *v* as the varying parameter.

Accordingly, we plot in Figure 9, for the Lipkin model (sub-index L), two things: (1) left: (β) versus $C_{f}\left(\beta \right)$ with N=12 and v=1; (2) center: F(N) versus $C_{f}\left(N\right)$ with β=10 and v=1; (3) right: F(v) versus $C_{f}\left(v\right)$ with N=12 and β=10.

**Figure 10:**
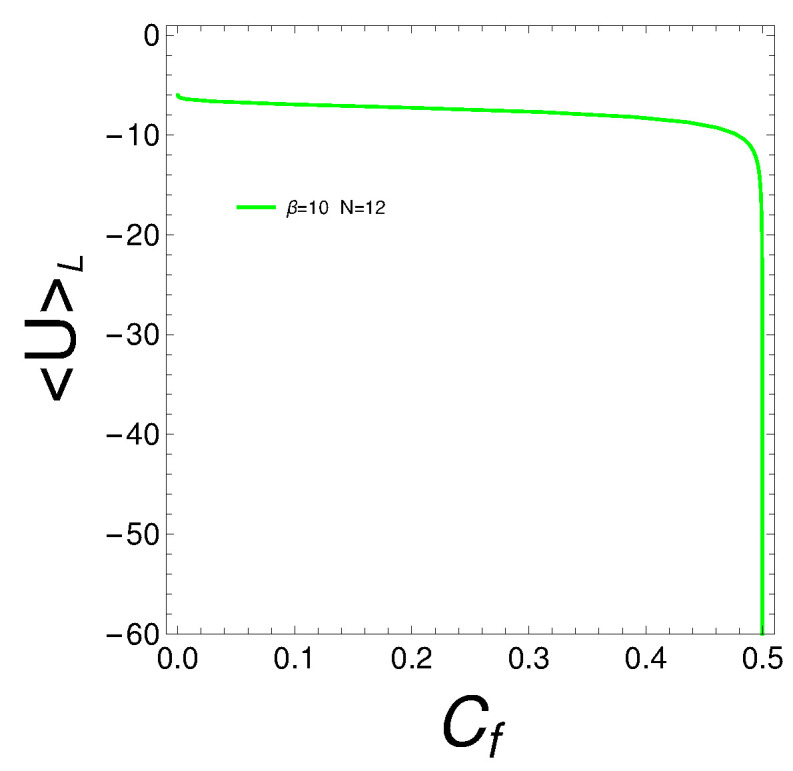
Lipkin’s mean energy $<U{>}_{L}$ plotted against the mixedness indicator $C_{f}$ for β=10.

The observation that the free energy becomes more negative with a larger degree of quantum mixedness in the Lipkin model can be attributed to increased entropy, lower effective energy, enhanced cooperative effects, and potential phase transitions. These factors collectively lead to a more energetically favorable state as the degree of quantum mixedness increases, thus lowering the free energy. See the plot of Figure 9. The increase in quantum mixedness can enhance correlations between particles. When these correlations reach a critical level, the system can reorganize into a more energetically favorable configuration. This reorganization would manifest itself as an abrupt decrease in the Lipkin mean energy $U_{L}$. This is what our $<U{>}_{L}$-plot in Figure 10 indeed shows. At a mixing of 0.5, the system seems to achieve a state of enhanced quantum coherence, where the superposition of states leads to a lower energy configuration. This coherence can stabilize the system, resulting in a sharp decrease in energy. The abrupt change could signify that the system is reaching an optimal balance between the different quantum states, minimizing energy through constructive interference and optimal state mixing. We next present a plot in Figure 10 for the man energy, which confirms some assertions made above.

## 6. Conclusions

This study elucidates the intricate dynamics of quantum mixing, particle number fluctuations, and temperature variations within the Lipkin model, revealing profound insights into the behavior of many-fermion systems. Our findings underscore several key points:

- The degree of quantum mixedness displays a strong dependency on the total number of fermions, highlighting distinct behaviors across different temperature regimes. This emphasizes the importance of considering fermion number in analyzing quantum systems, as it directly influences the system’s mixedness and overall state.
- Remarkably, the observed dependencies of quantum mixedness on fermion number and temperature are robust against variations in the fermion–fermion interaction strength, provided the interaction is non-zero. This robustness suggests that the fundamental properties of quantum mixedness are intrinsic to the system’s structure rather than being heavily influenced by interaction specifics.
- The insights gained from this study have potential implications for various fields, including condensed matter physics and quantum information science. Understanding the dependencies and behaviors of quantum degree of mixedness in fermionic systems can inform the development of quantum technologies and enhance the theoretical models used to describe complex quantum systems.

Through a comprehensive numerical exploration of key features of the system, we have provided a detailed analysis of the Lipkin model, offering valuable perspectives on the fundamental characteristics of many-fermion systems. Our results contribute to a deeper understanding of quantum phenomena in fermionic systems, paving the way for future research and practical applications in related fields.

## Data Availability

The original contributions presented in this study are included in the article.
